# Steep right anterior oblique view of self-expandable transcatheter aortic valve to timely detect stent under-expansion or non-uniform expansion before final release: a case series

**DOI:** 10.1093/ehjcr/ytae405

**Published:** 2024-08-07

**Authors:** Umihiko Kaneko, Daisuke Hachinohe, Ken Kobayashi, Hidemasa Shitan, Ryo Horita, Ryo Ootake, Tsutomu Fujita

**Affiliations:** Department of Cardiovascular Medicine, Sapporo Cardio Vascular Clinic, North 49, East 16, 8-1 Higashi Ward, Sapporo, Hokkaido 007-0849, Japan; Department of Cardiovascular Medicine, Sapporo Cardio Vascular Clinic, North 49, East 16, 8-1 Higashi Ward, Sapporo, Hokkaido 007-0849, Japan; Department of Cardiovascular Medicine, Sapporo Cardio Vascular Clinic, North 49, East 16, 8-1 Higashi Ward, Sapporo, Hokkaido 007-0849, Japan; Department of Cardiovascular Medicine, Sapporo Cardio Vascular Clinic, North 49, East 16, 8-1 Higashi Ward, Sapporo, Hokkaido 007-0849, Japan; Department of Cardiovascular Medicine, Sapporo Cardio Vascular Clinic, North 49, East 16, 8-1 Higashi Ward, Sapporo, Hokkaido 007-0849, Japan; Department of Cardiovascular Medicine, Sapporo Cardio Vascular Clinic, North 49, East 16, 8-1 Higashi Ward, Sapporo, Hokkaido 007-0849, Japan; Department of Cardiovascular Medicine, Sapporo Cardio Vascular Clinic, North 49, East 16, 8-1 Higashi Ward, Sapporo, Hokkaido 007-0849, Japan

**Keywords:** Case report, Non-uniform expansion, Steep RAO view, Stent under-expansion, Transcatheter aortic valve replacement, Valve embolization

## Abstract

**Background:**

Severely calcified aortic valves are a major limitation of transcatheter aortic valve replacement, because eccentric and heavy calcification of the aortic valve occasionally inhibits self-expansion of the valve frame, resulting in stent under-expansion, including non-uniform expansion or infolding. Nevertheless, the two-dimensional nature of fluoroscopic projection imaging can limit detection of stent under-expansion prior to the final release.

**Case summary:**

We present two cases demonstrating the importance of the steep right anterior oblique (RAO) view (>50°) in detecting significant stent under-expansion of a self-expanding valve prior to the final release. In Case 1, despite enough pre-dilatation, the partially deployed transcatheter heart valve (THV) appeared to be a substantial under-expansion, which was detected only in steep RAO view. Immediately after the final release, the THV was spontaneously embolized into the ascending aorta (the so-called ‘pop-up’ phenomenon). Emergent implantation of balloon-expandable valve proved to be successful as a bailout. In Case 2, significant stent distortion and infolding, especially on the non-coronary cusp side, was successfully diagnosed only in a steep RAO view prior to the final release. This finding allowed THV recapture and replacement with a new THV.

**Discussion:**

Notably, the steep RAO view can visualize the THV from its short axis while eliminating parallax, allowing for accurate diagnosis of THV under-expansion particularly in patients with severe calcification in the non- or right-coronary cusp. Therefore, the steep RAO view allows timely detection of THV under-expansion before the final release, enabling THV recapture and the adoption of several management strategies.

**Learning points**

- Undetected/untreated under-expansion or non-uniform expansion of a self-expandable transcatheter heart valve (THV) prior to the final release may result in THV embolization and migration (pop-up phenomenon).
- The conventional cusp-overlap [right anterior oblique (RAO) caudal] and left anterior oblique views have a limited range of observation, making it difficult to detect THV under-expansion. Because the steep RAO view (>50°) can visualize the THV from its short axis, parallax is eliminated and under-expansion can be precisely detected. This allows for THV recapture and the implementation of various management strategies.

## Introduction

Stent under-expansion, including non-uniform expansion (NUE) or the infolding phenomenon induced by severely calcified aortic valves, is a major limitation of transcatheter aortic valve replacement (TAVR), even with the latest generation of self-expanding transcatheter heart valves (THVs). The cusp-overlap [right anterior oblique (RAO) caudal] and left anterior oblique (LAO) (three-cusp coplanar) views are frequently used as the primary fluoroscopic angles for assessment of implantation depth.^1^ Nevertheless, these two angles are not always the best views to detect significant under-expansion of the THV, because overlapping of the stent frame can prevent visualization from the short axis of the THV and limit the detection of stent under-expansion. We present two cases that demonstrate the utility of the steep RAO (>50°) view for detecting significant under-expansion of the THV prior to complete release, as well as a simplified and optimized fluoroscopic viewing strategy.

## Summary figure

**Figure:**
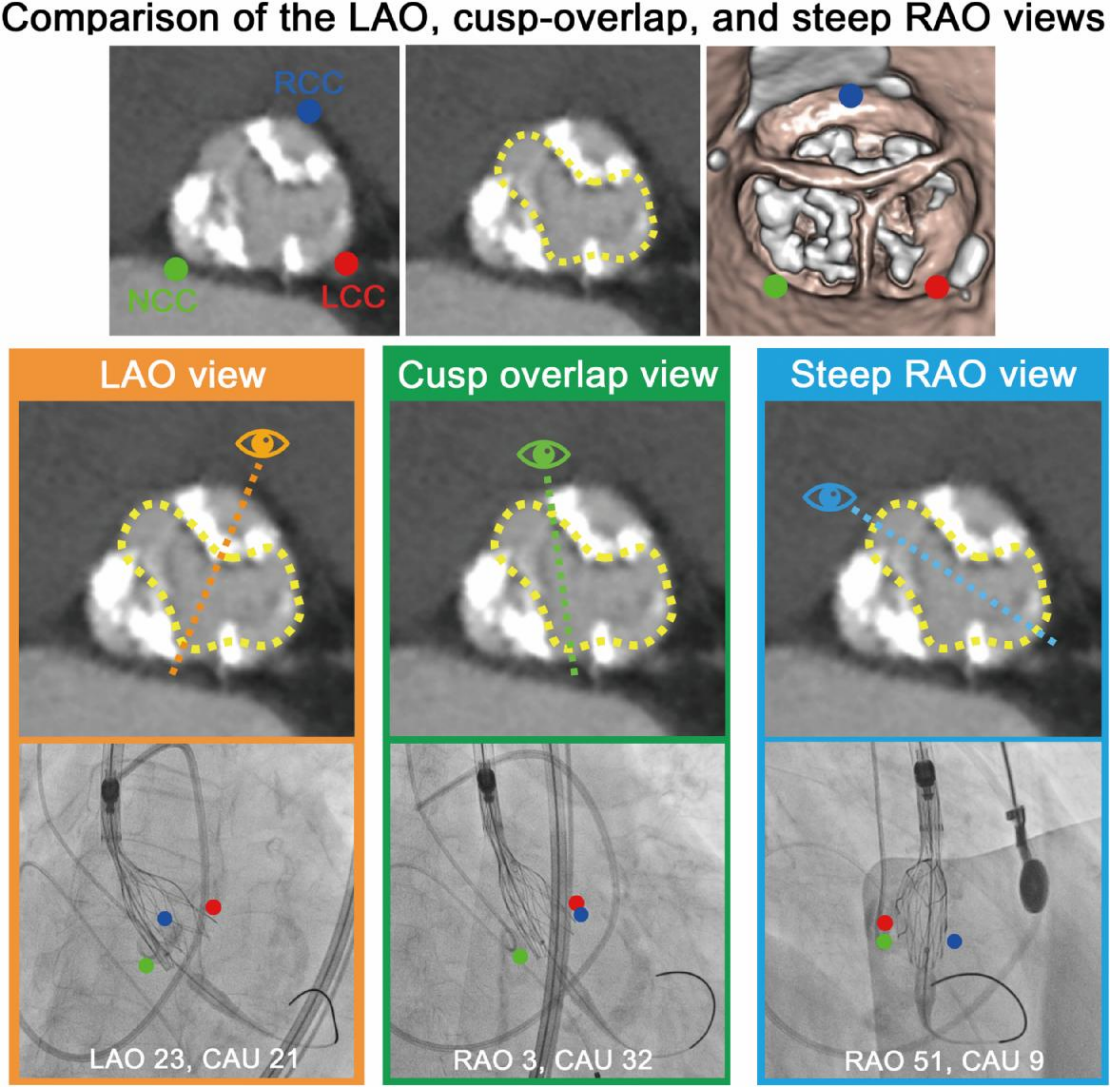


## Case presentation

### Patient 1

**Figure 1:**
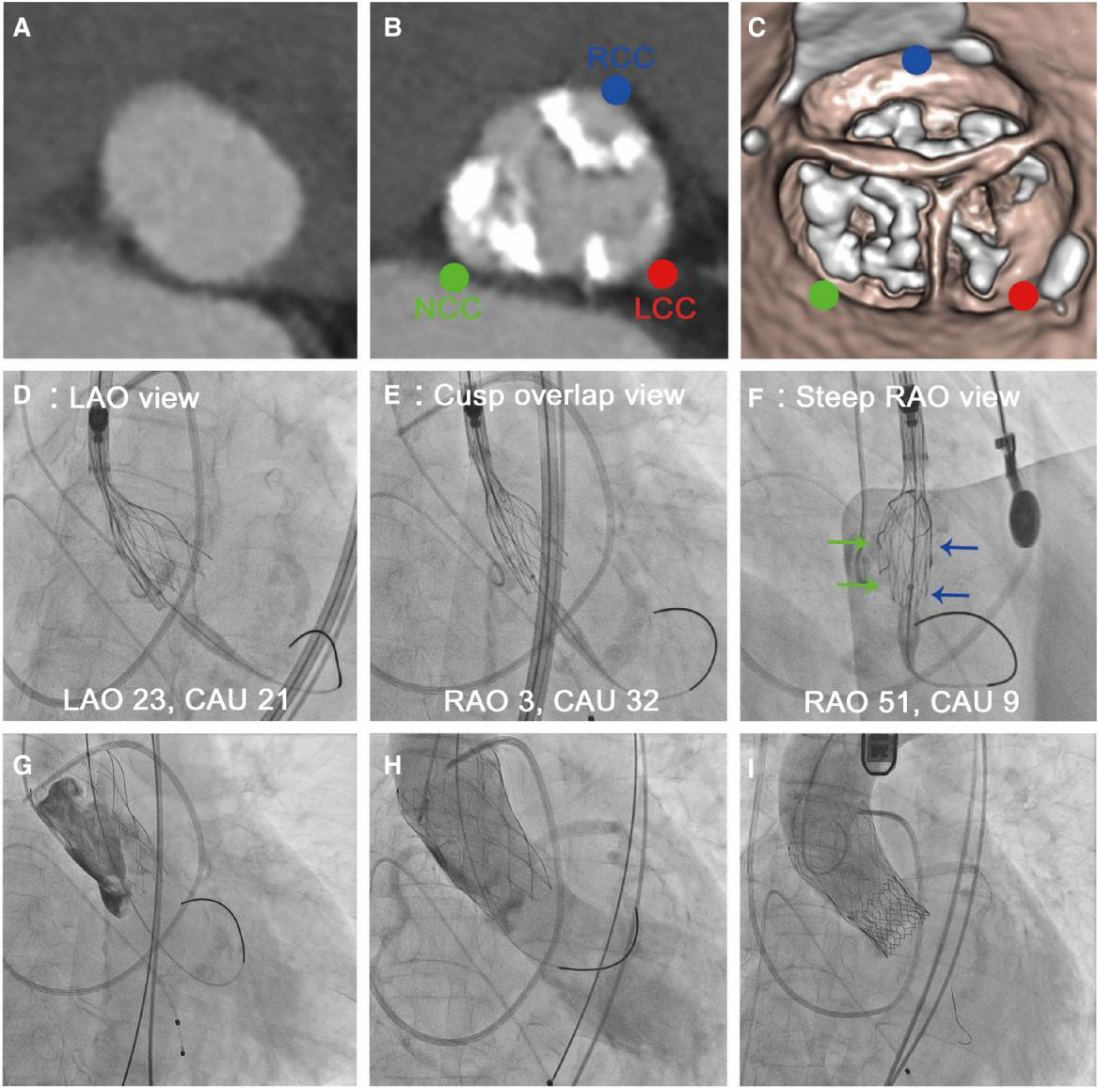
Significant transcatheter heart valve under-expansion leading to embolization. (*A–C*) Pre-procedural computed tomography imaging showing a heavily calcified aortic valve, especially in the right- and non-coronary cusps. (*A*) Aortic annulus. (*B*) 6 mm above the annulus. (*C*) Volume-rendering image. (*D* and *E*) In the left anterior oblique and cusp-overlap views, the transcatheter heart valve seemed to be well expanded. (*F*) Only the steep right anterior oblique view elucidated a constrained transcatheter heart valve at 80% deployment. Significant transcatheter heart valve under-expansion is indicated by green arrows in the non-coronary cusp and blue arrows in the right-coronary cusp. (*G*) Transcatheter heart valve embolization just after the final release. (*H*) Severe aortic regurgitation. (*I*) Emergent implantation of a balloon-expandable valve proved successful as a bailout. The blue circle represents the right-coronary cusp; the red circle represents the left-coronary cusp; and the green circle indicates the non-coronary cusp.

An 84-year-old female with symptomatic severe aortic stenosis (AS) was scheduled to undergo TAVR using a 27-mm Navitor self-expanding valve (SEV) (Abbott, USA) under local anaesthesia. Pre-procedural computed tomography (CT) revealed a significantly calcified and tricuspid aortic valve (calcium score, 2503 AU) with an aortic annulus area of 425 mm^2^, perimeter of 75.1 mm, maximum diameter of 27.6 mm, and minimum diameter of 18.9 mm (*Figure 1A–C*). A heavy calcification was distributed especially in the right-coronary cusp (RCC) and non-coronary cusp (NCC) (*Figure 1B* and *C*). After pre-dilatation with an 18-mm balloon and partial release of a 27 mm Navitor valve, the cusp-overlap view (RAO: 3°/caudal: 32°) and the LAO view (LAO: 23°/caudal: 21°) both appeared to show a well-expanded THV, but only the steep RAO view (RAO: 51°/caudal: 9°) indicated the restricted THV at 80% deployment. After recapture, further pre-dilatation with a 20-mm balloon yielded full expansion, and the THV was partially deployed. The THV appeared to be substantially expanded and well seated in both the cusp-overlap and LAO views (*Figure 1D* and *E*). Notable THV under-expansion or NUE that had been overlooked was only detected in the steep RAO view (*Figure 1F*). Under the suspicion of NUE, recapture and replacement with a new THV were performed. However, only the steep RAO view revealed apparent NUE again. The valve was eventually deployed sufficiently deep (i.e. 4 mm below the annulus) in preparation for post-dilatation following the final release. Immediately after its final release, the THV became unstable in its deployment position and eventually embolized into the ascending aorta (*Figure 1G* and *H*). The patient experienced cardiogenic shock owing to severe aortic regurgitation. Under left coronary protection, emergent implantation of balloon-expandable valve (26-mm SAPIEN 3 Ultra RESILIA; Edwards Lifesciences LLC, Irvine, CA, USA) proved to be successful as a bailout (*Figure 1I*).

### Patient 2

**Figure 2:**
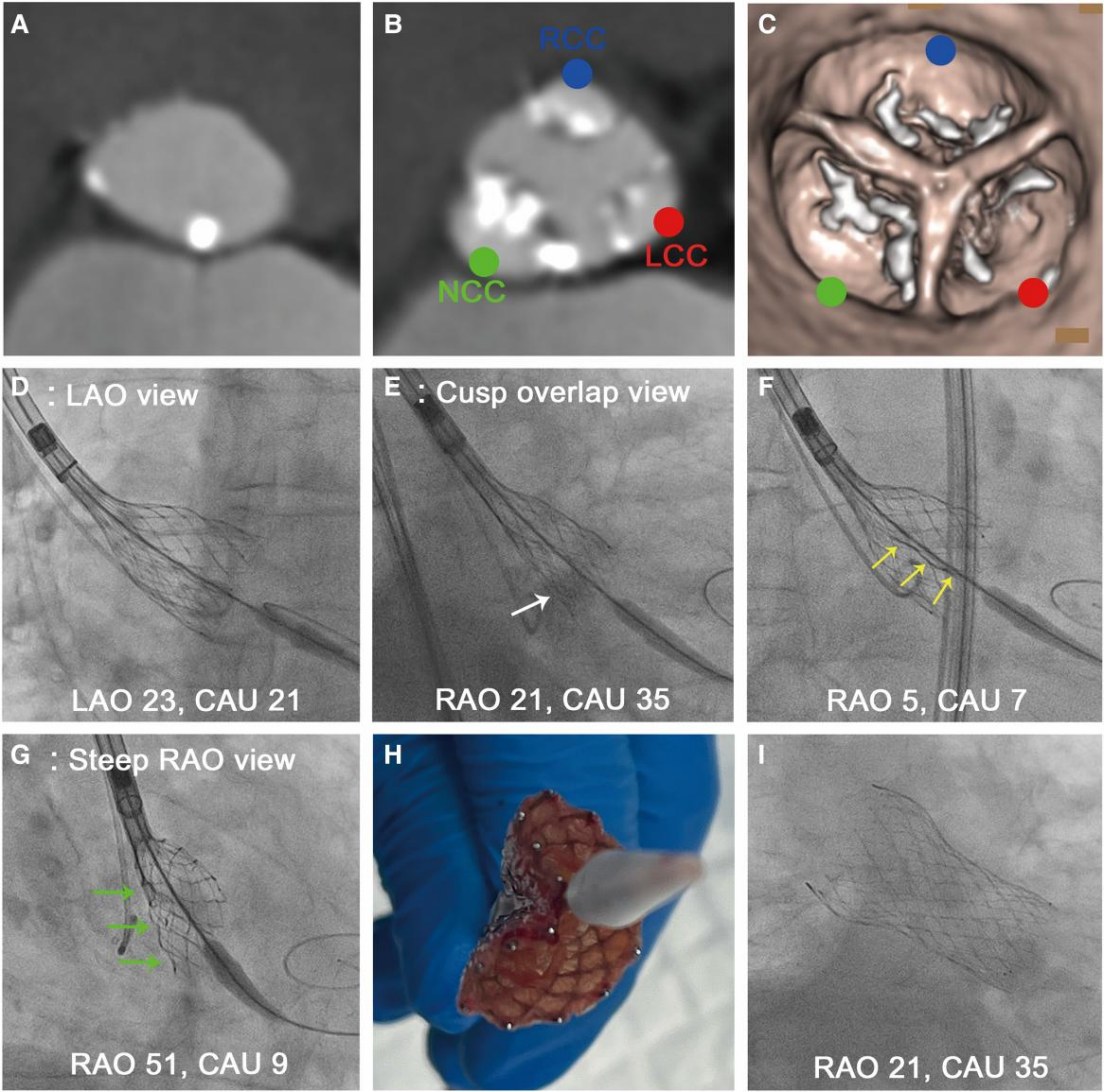
Transcatheter heart valve infolding diagnosed in the steep right anterior oblique view. (*A–C*) Baseline computed tomography imaging showing a heavily calcified aortic valve, especially in the non-coronary cusp. (*A*) Aortic annulus. (*B*) 6 mm above the annulus. (*C*) Volume-rendering image. (*D*) The transcatheter heart valve appeared to be well expanded in the left anterior oblique view. (*E*) In the cusp-overlap view, it appeared to be slightly under-expanded (white arrow). (*F*) Using rotational fluoroscopy, a vertical line along the valve frame was suspected (yellow arrows). (*G*) Significant stent distortion, particularly in the non-coronary cusp side, was first identified in the steep right anterior oblique view (green arrows). (*H*) Infolded transcatheter heart valve. (*I*) A new transcatheter heart valve was successfully implanted with full expansion. The blue circle represents the right-coronary cusp; the red circle represents the left-coronary cusp; and the green circle indicates the non-coronary cusp.

A 96-year-old man with symptomatic severe AS and a history of heart failure underwent transfemoral TAVR. Pre-operative CT showed heavily calcified leaflets of the aortic valve (calcium score, 2577 AU) with an aortic annulus area of 490 mm^2^, perimeter of 80.9 mm, maximum diameter of 29.6 mm, and minimum diameter of 21.4 mm (*Figure 2A–C*). A heavy calcification was located mainly on the NCC (*Figure 2B* and *C*). Following pre-dilatation with a 20-mm balloon without indentation, 29-mm self-expanding Evolut FX (Medtronic, Minneapolis, MN, USA) was partially deployed in the optimal position. The THV seemed to be appropriately expanded in the LAO view (LAO: 32°/caudal: 6°), but appeared to be slightly under-expanded in the cusp-overlap view (RAO: 21°/caudal: 35°) (*Figure 2D* and *E*). Rotational fluoroscopy indicated a vertical line along the valve frame (*Figure 2F*). After switching to a steep RAO view (RAO: 51°/caudal: 9°), significant stent distortion, notably on the NCC side, was correctly diagnosed (*Figure 2G*). After recapturing and retrieving the THV, an infolded THV with significant invagination of the frame was detected (*Figure 2H*). Further pre-dilatation with a 22-mm balloon and replacement with a second 29-mm Evolut FX were successful, with excellent stent expansion confirmed by multidirectional fluoroscopy (*Figure 2I*).

## Discussion

These two cases presented herein highlight the following important findings: (i) only the steep RAO view, and neither the cusp-overlap nor the LAO views, can diagnose substantial stent under-expansion, including NUE or infolding in patients with heavy and eccentric calcification, and (ii) under-expansion or NUE of an SEV can be the main cause of THV embolization into the ascending aorta just after the final release (also known as the ‘pop-up’ phenomenon).

During TAVR procedures utilizing SEVs, the cusp-overlap and LAO views are frequently used as the primary fluoroscopic views. These views are essential for precise assessment of implantation depth, thereby mitigating the risk of interaction with the conduction system and contributing to the prevention of conduction disturbances.^1,2^ In a recent report, the NCC and RCC are the main locations of under-expanded stent frames, where early THV stent frame expansion occurs and substantial calcification is present.^3^ Notably, our report highlighted that the specific fluoroscopic findings of THV under-expansion could be potentially overlooked when only the cusp-overlap and LAO views are used due to the two-dimensional nature of fluoroscopic projection imaging. An additional solution would involve the use of steep RAO view and rotating fluoroscopic analysis (projections from the RAO view to the LAO view).^4^

Visualizing the short axis of the aortic valve annulus allows for a more precise evaluation of THV expansion, whereas the LAO view primarily focus on the long axis of the annulus. According to a previous report, CT-defined mean projection angle of cusp-overlap view was RAO 12.9 ± 12.5 and caudal 26.9 ± 10.4 degrees, and coplanar (LAO) view was LAO 9.4 ± caudal 10.8 degrees.^5^ These data suggests that fluoroscopy from the cusp-overlap and LAO views may create *large blind spots*, especially at steep RAO angles (defined as >50°). To compensate for these large blind spots, the additional use of a steep RAO view is highly recommended for displaying the short axis of the THV. The most significant constraint of the THV is best appreciated in this specific view because it can image the short axis of the elliptical aortic annulus, especially with severe calcification in the NCC or RCC (*Figures 1F* and *2G*).

**Figure 3:**
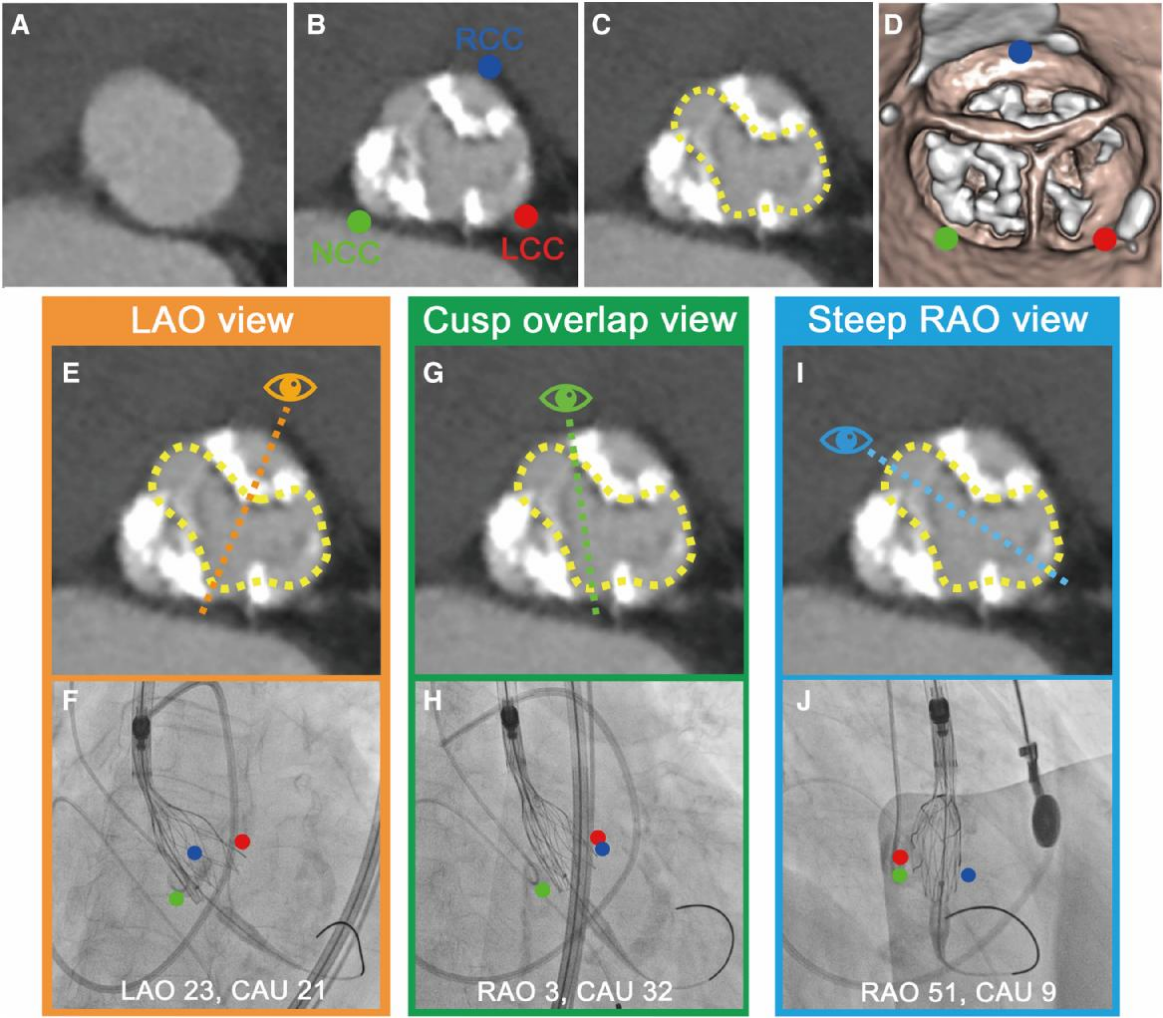
Comparison of the left anterior oblique, cusp-overlap, and steep right anterior oblique views. (*A–D*) Pre-procedural computed tomography imaging showing heavy calcification, especially in the right- and non-coronary cusps. (*A*) Baseline annulus. (*B*) Uneven distribution of heavy calcification in the three cusps. (*C*) Theoretically predicted shape of a transcatheter heart valve that is partially deployed and under-expanded (yellow-dotted line). (*D*) Volume-rendering image. (*E* and *F*) Left anterior oblique view observed by an operator in the direction of the dotted orange line. (G and *H*) An operator views the cusp-overlap view towards the dotted green line. (*I*) Steep right anterior oblique view observed by an operator in the direction of the dotted blue line. (*J*) Only the steep right anterior oblique view elucidates the constrained transcatheter heart valve. The blue circle represents the right-coronary cusp; the red circle represents the left-coronary cusp; and the green circle indicates the non-coronary cusp.

In Patient 1, fluoroscopic evaluation from a steep RAO view confirmed that the partially deployed valve had a distorted shape and apparent NUE due to eccentric and heavy calcification in the NCC and RCC (*Figure 1F*). Because conventional cusp-overlap and LAO projections are viewed by physicians with a greater projection along the annular long axis, the stent frame appeared to overlap, causing failure of visualization from the short axis of the constrained THV and overlooking NUE (*Figure 3A–H*). Notably, the steep RAO projection allows the constrained THV to be displayed more towards the short axis of the THV and enables accurate detection of under-expansion (*Figure 3I* and *J*; dotted blue lines).

While spontaneous embolization of a SEV into the ascending aorta (‘pop-up’ phenomenon) is a fatal complication that can cause aortic dissection, or coronary obstruction, literature on the precise mechanism is scarce.^6–9^ In Patient 1, owing to insufficient opening force and radial outward force to fully expand against the severely calcified aortic valve, the stent frame significantly under-expanded prior to the final release. Therefore, just after the final release, the THV was unable to expand further at the deployed position and was gradually extruded because of heavy calcification, finally leading to embolization into the ascending aorta. Notably, our report highlights the significance of THV under-expansion before the final release as the main cause of THV embolization.

**Figure 4:**
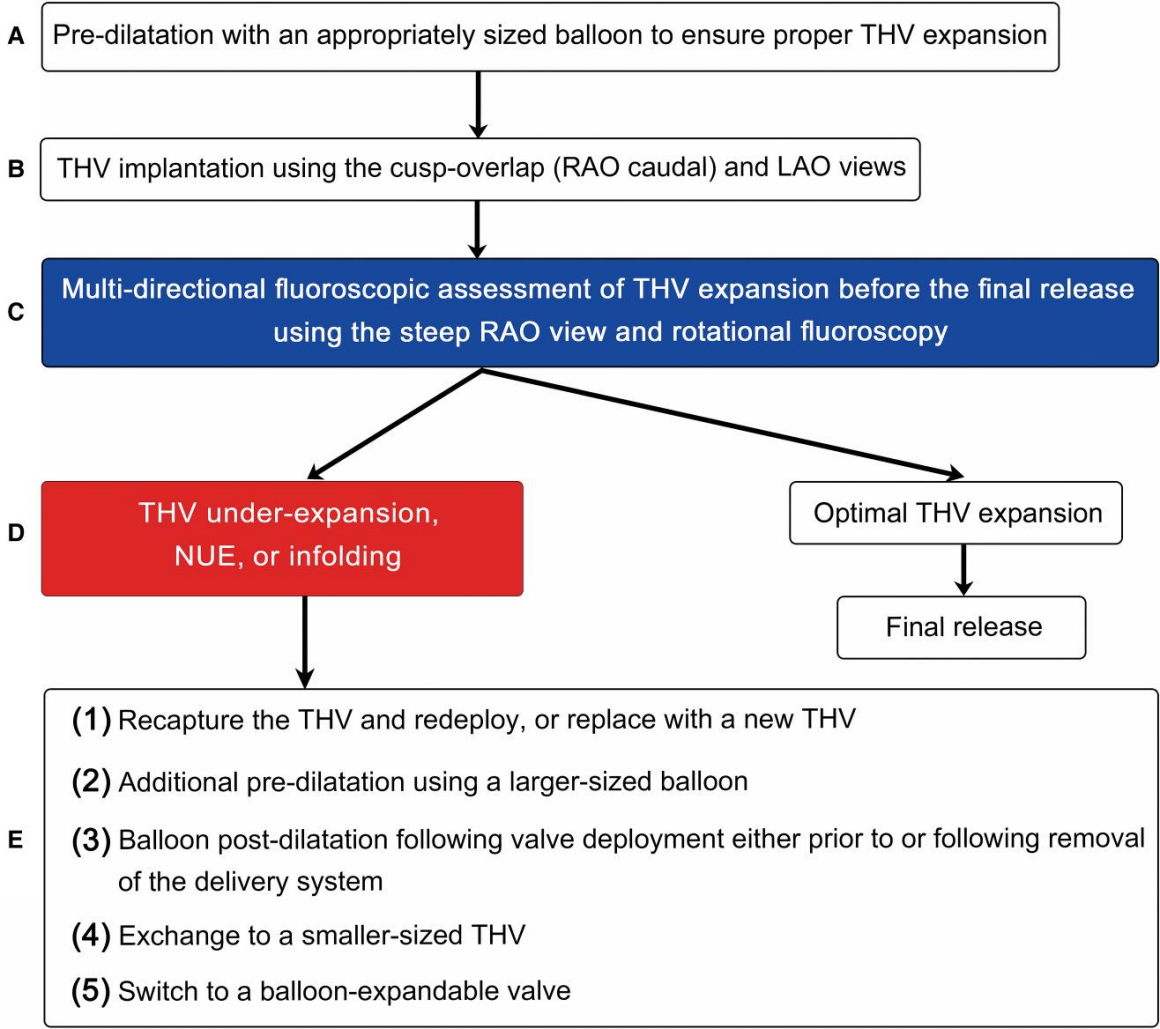
Algorithm for addressing heavy and eccentric calcified aortic valves. LAO, left anterior oblique; NUE, non-uniform expansion; RAO, right anterior oblique; THV, transcatheter heart valve.

When regional THV under-expansion is identified before the final release, the following additional strategies can be employed to overcome the problem: (i) recapturing and redeployment or replacement with a new THV; (ii) additional pre-dilatation using a larger-sized balloon; (iii) balloon post-dilation following valve deployment either prior to or following removal of the delivery system; (iv) exchanging to a smaller-sized valve; and (v) switching to a balloon-expandable valve (*Figure 4*).^10–16^

In conclusion, the steep RAO view may be the best fluoroscopic viewing angle for accurate detection of stent under-expansion in patients showing heavy and eccentric calcifications in the NCC and RCC, since the short axis of the constrained THV can be precisely visualized only from this view. This report elucidates the undetected/untreated under-expansion or NUE as the primary cause of THV embolization and migration (pop-up phenomenon) immediately after release. Therefore, early detection of significant stent under-expansion with multidirectional fluoroscopic assessment of the THV, including the steep RAO view before complete release, is crucial, allowing THV recapture and the implementation of several management strategies.

## Lead author biography


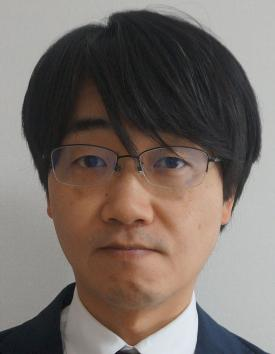


Dr Umihiko Kaneko is a clinical cardiologist who graduated from Tohoku University (Sendai, Japan) in 2003. He currently serves as a director of the interventional cardiology at Sapporo Cardiovascular Clinic (Sapporo, Japan). His primary area of expertise includes interventional cardiology and TAVR. His heart team has published several papers on TAVR techniques.

## Data Availability

The data underlying this article are available in the article.
